# Rates of Future Lumbar Fusion in Patients with Cauda Equina Syndrome Treated With Decompression

**DOI:** 10.5435/JAAOSGlobal-D-22-00153

**Published:** 2022-11-02

**Authors:** Henry D. Seidel, Sean Pirkle, Sarah Bhattacharjee, Hayden P. Baker, Michael J. Lee, Mostafa H. El Dafrawy

**Affiliations:** From the Pritzker School of Medicine (Mr. Seidel and Dr. Bhattacharjee), University of Chicago, Chicago, IL; the Department of Orthopaedics and Sports Medicine (Dr. Pirkle), University of Washington, Seattle, WA; and the Department of Orthopaedic Surgery and Rehabilitation Medicine (Dr. Baker, Dr. Lee, and Dr. El Dafrawy), University of Chicago, Chicago, IL.

## Abstract

The long-term risk of conversion to lumbar fusion is ill-defined for patients with cauda equina syndrome (CES) treated with decompression. This study aimed to identify the rates of fusion in patients with CES and compare those rates with a matched lumbar spinal stenosis (LSS) group.

**Methods:**

Patients with CES who underwent decompression were identified in a national database and matched to control patients with LSS. The rates of conversion to fusion were identified and compared. Multivariate logistic regression analysis identified independently associated risk factors. A subanalysis was conducted after stratifying by timing between CES diagnosis and decompression.

**Results:**

The rate of lumbar fusion in the CES cohort was 3.6% after 1 year, 6.7% after 3 years, and 7.8% after 5 years, significantly higher than the LSS control group at all time points (1 year: 1.6%, *P* = 0.001; 3 years: 3.0%, *P* < 0.001; 5 years: 3.8%, *P* < 0.001). CES was independently associated with increased risk of conversion to fusion (odds ratio: 2.13; 95% confidence interval: 1.56 to 2.97; *P* < 0.001). Surgical timing was not associated with risk of conversion to fusion.

**Conclusions:**

After 5 years, 7.8% of patients with CES underwent fusion, a markedly higher rate compared with patients with LSS. Counseling patients with CES on this increased risk of future surgery is important for patient education and satisfaction.

Cauda equina syndrome (CES) is a rare but serious condition that results from compression of the lumbar and sacral nerve roots. The most common cause is lumbar disk herniation, although a variety of etiologies, including spinal neoplasm, abscess, epidural hematoma, and trauma, have been reported.^1,2,3,4,5,6^ Patients experiencing CES often present with an array of symptoms, including low back pain; lower extremity weakness; perianal hypoesthesia; and bowel, bladder, or sexual dysfunction.^7–9^

Management of CES centers around decompression of the spinal nerve roots. CES caused by disk herniation is commonly treated with laminectomy and diskectomy at the level of the herniation; however, a number of decompression techniques can be conducted, varying by the specific compressive pathology.^7,10,11^ A full or wide laminectomy with generous bone removal is often conducted in patients with CES,^12,13^ as compared with a partial laminectomy for uncomplicated disk herniation or lumbar spinal stenosis (LSS). The timing of decompression for CES is also of considerable interest in the literature. The generally accepted view is that decompression should be conducted within 48 hours of the onset of symptoms because multiple studies have demonstrated improved neurological outcomes when treatment occurs within this indicated window.^12,14,15,16,17,18,19^ Thus, these potentially challenging operations are often conducted on an urgent basis with less-than-optimal conditions.

Although concomitant lumbar fusion may be conducted during the index decompression procedure, most patients with CES are treated with decompression alone.^20^ The long-term rates of conversion to fusion in this patient population are not clear because most of the clinical studies investigating postoperative outcomes in CES have focused on the resolution of neurological deficits.^15,21,22,23,24^ Requiring additional surgery may result in increased morbidity for these patients; thus, accurate prognostic information regarding the risk of conversion to lumbar fusion is important for patient counseling.

The goal of this study was to define the rates of future lumbar fusion in patients with CES managed with decompression alone at the time of initial presentation and compare those rates with a matched LSS group. Furthermore, we sought to investigate how the timing between CES diagnosis and decompression surgery affected long-term risk of conversion to fusion.

## Methods

### Database

We conducted a retrospective study of patients from the Mariner national insurance claims database, available through PearlDiver (PearlDiver), consisting of anonymized records from 53 million orthopaedic patients between the years 2010 to 2018. The Mariner database contains insurance claims from multiple payer types, including commercial insurance, Medicare, Medicaid, and self-pay. It is searchable by billing codes identified in the database with Current Procedural Terminology (CPT) codes and International Classification of Diseases, Ninth Revision (ICD-9) and 10^th^ Revision (ICD-10) diagnostic and procedural codes. All records in PearlDiver are Health Insurance Portability and Accountability Act (HIPAA) compliant. This study was determined exempt from additional review by our Institutional Review Board.

### Patient Selection and Outcomes

Patients who underwent decompression within 1 month of a diagnosis of CES were identified in the database using CPT, ICD-9, and ICD-10 codes (Appendix, http://links.lww.com/JG9/A244). To account for patients who changed or lost insurance or left the database for other reasons, patients were excluded if they did not have continuously active records in the database over the study period; thus, all patients had a 5-year follow up in this study. Patients in the CES cohort were matched to patients from a LSS control group. The control group underwent similar decompression procedures as the CES group but lacked a diagnosis of CES and instead had an ICD-9 or ICD-10 code specific for LSS (Appendix, http://links.lww.com/JG9/A244). To further characterize the patients in the LSS control group, we identified the percentage of patients with an associated diagnosis of facet hypertrophy or disk extrusion. This LSS control group was matched in a 1:1 ratio with the CES cohort by the matching parameters of age, sex, obesity, tobacco use, and diabetes. Patients who underwent lumbar fusion on the same day as the index decompression procedure were excluded from the CES cohort and LSS control group. The 1-year, 3-year, and 5-year rates of conversion to fusion, as defined by a CPT or ICD procedure code for lumbar fusion (Appendix, http://links.lww.com/JG9/A244), were identified for the two groups.

### Statistical Analysis

The Pearson chi squared test was used to assess the univariate differences in rates of conversion to lumbar fusion 1, 3, and 5 years after decompression surgery between the CES cohort and LSS control group. Differences between the 2 groups were determined to be notable at an alpha level of 0.05. To further investigate the independent association between CES and conversion to lumbar fusion 5 years after the index decompression surgery, a multivariate logistic regression analysis was conducted, accounting for the variables of CES diagnosis, age, sex, obesity, diabetes, and tobacco use because these are commonly investigated demographic and comorbidity risk factors associated with spinal revision surgery procedures.^25–27^ Adjusted odds ratios (ORs) and confidence intervals (CIs) were determined. Statistical analysis was conducted using the R statistical package available within PearlDiver software.

### Timing to Decompression Subanalysis

A subanalysis based on the timing of decompression was additionally conducted. Patients in the CES cohort were stratified into three subgroups: <48 hours, 48 hours—10 days, and 10 days—30 days between CES diagnosis and decompression. The timing of these subgroups was chosen because 48 hours is the classically recommended window for intervention in patients diagnosed with CES; furthermore, previous studies have used similar timing cutoffs.^15,16,28^ We identified the 5-year rates of conversion to lumbar fusion of patients from these three subgroups. Univariate logistic regression analysis was used to determine differences in the rates of conversion to lumbar fusion between the three subgroups.

## Results

### Patient Identification and Matching

A total of 1800 patients who underwent decompression surgery after a CES diagnosis with a 5-year follow-up in the database were identified. Of these patients, 1288 (71.6%) received decompression alone while 512 (28.4%) underwent lumbar fusion in addition to decompression. The patients treated with decompression alone made up the CES cohort of this study and were matched to 1288 control patients who underwent decompression for LSS. Among the patients in the LSS control group, 34.0% (438) had associated disk extrusion billing codes, 7.0% (90) had facet hypertrophy codes, 49.2% (634) had both codes, and 9.8% (126) did not have a billing code of disk extrusion or facet hypertrophy associated and underwent decompression because of another etiology of LSS. The CES cohort and LSS control groups consisted of patients with similar distributions of sex, diabetes and obesity comorbidity, and tobacco use (Table 1). The age of patients in the CES cohort (53.6 ± 15.9) was observed to be higher than those in the LSS control group (52.0 ± 15.5) even after matching because of limited availability of control patients (*P* = 0.010).

**Table 1:** Demographics and Comorbidities of Patients Compared Between the Matched CES Cohort and Non-CES LSS Control Group

Characteristic	CES Cohort (N = 1288)	LSS Control Group (N = 1288)	*P*
Age, mean ± SD	53.6 ± 15.9	52.0 ± 15.5	0.010
Female, % (n)	48.0% (618)	48.0% (618)	1.000
Male, % (n)	52.0% (670)	52.0% (670)	1.000
Diabetes, % (n)	45.1% (581)	45.1% (581)	1.000
Obesity, % (n)	49.5% (637)	49.5% (637)	1.000
Tobacco use, % (n)	32.1% (413)	32.1% (413)	1.000

CES = cauda equina syndrome, LSS = lumbar spinal stenosis

### Outcome Measures

**Figure 1:**
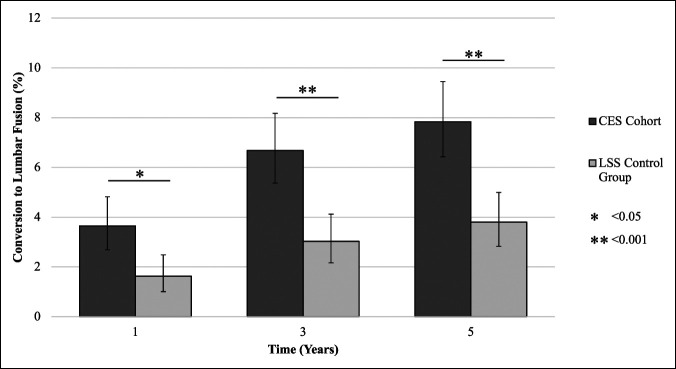
Graph showing long-term rates of conversion to fusion after index decompression surgery compared between patients with CES and patients who underwent decompression for LSS not associated with CES. Error bars represent 95% confidence intervals. CES = cauda equina syndrome, LSS = lumbar spinal stenosis

3.6% of patients in the CES cohort required lumbar fusion after 1 year, 6.7% after 3 years, and 7.8% after 5 years (Figure 1). Patients in the LSS control group underwent conversion to lumbar fusion at the rates of 1.6% after 1 year, 3.0% after 3 years, and 3.8% after 5 years. Univariate analysis identified the rates to be significantly higher among the patients with CES at all time points (*P* = 0.001 at 1 year; *P* < 0.001 at 3 and 5 years).

### Multivariate Analysis

Multivariate logistic regression analysis identified a diagnosis of CES as independently associated with an increased risk of 5-year conversion to lumbar fusion (OR: 2.13; 95% CI: 1.56 to 2.97; *P* < 0.001; Table 2). Male sex (OR: 0.46; CI: 0.35 to 0.60; *P* < 0.001) and age 65 years or older (OR: 0.57; CI: 0.40 to 0.78; *P* < 0.001) were associated with decreased risk of conversion to lumbar fusion at 5 years after decompression surgery. Obesity, tobacco use, and diabetes were not found to be markedly associated with 5-year conversion to lumbar fusion.

**Table 2:** Multivariate Logistic Regression Analysis of 5 Year Rate of Conversion to a Fusion Procedure After Index Decompression

Variables	Odds Ratio (95% Confidence Interval)	*P*
Cauda equina syndrome	2.13 (1.56-2.97)	**<0.001**
Sex (male)	0.46 (0.35-0.60)	**<0.001**
Age older than 65 years	0.57 (0.40-0.78)	**<0.001**
Obesity	1.27 (0.97-1.66)	0.085
Tobacco use	1.25 (0.95-1.64)	0.103
Diabetes	1.10 (0.84-1.43)	0.500

Bolded entries indicate statistical significance.

### Timing to Decompression Subanalysis

Of the 1288 patients who made up the CES cohort, 1118 (86.8%) underwent decompression within 48 hours of CES diagnosis, 94 (7.3%) between 48 hours and 10 days, and 76 (5.9%) between 10 days and 1 month. The 5-year rates of conversion to lumbar fusion were 8.0%, 5.3%, and 9.2%, respectively. Although the rates trended higher in the <48 hour and 10 days—1-month groups, in univariate logistic regression analysis, the timing of decompression showed no notable effect on 5-year rate of conversion to lumbar fusion (Table 3).

**Table 3:** Five-Year Rates of Conversion to Fusion Stratified by Timing Between Cauda Equina Syndrome Diagnosis and Decompression Surgery

Timing Subgroups (n)	5-Year Conversion to Fusion, % (n)	Odds Ratio (95% Confidence Interval)	*P*
<48 hr (1,118)	8.0% (89)	1.13 (0.62-2.11)	0.710
48 hr to 10 d (94)	5.3% (5)	0.65 (0.26-1.64)	0.360
10 d to -30 d (76)	9.2% (7)	1.22 (0.54-2.73)	0.629

## Discussion

Understanding the long-term outcomes of CES treated with decompression is important for patient counseling, setting reasonable postoperative expectations, and the future care of these patients. Although various outcomes related to CES have been reported, the risk of conversion to fusion after decompression laminectomy for CES is largely unknown. In this study, we observed the rate of conversion to lumbar fusion to be markedly higher at 1 year, 3 years, and 5 years between patients with CES and control patients with LSS who underwent decompression alone at the index procedure. In addition, in multivariate analysis, we found CES to be independently associated with a 5-year rate of conversion to lumbar fusion after controlling for patient demographic and relevant comorbidity factors. In a subanalysis of timing from CES diagnosis to decompression, most of the patients (86.8%) were treated within 48 hours; however, no significance was found between the future risk of conversion to lumbar fusion compared between the timing groups.

Currently, apart from reports that decompression surgeries for CES, which often involve a wide or complete laminectomy, may result in instability, the literature is scant regarding risk of future conversion to fusion.^20,29^ To the best of our knowledge, this is the largest study to date investigating the risk of future lumbar fusion after decompression alone in patients with CES. In studies not associated with CES, the reported risk of conversion to fusion is poorly defined for patients treated with decompression alone for LSS and degenerative disk disease. Most studies have sought to investigate revision surgery rates after laminectomy, which may include repeat decompression or conversion to fusion. A recent systematic review by Lang et al.^30^ reported the 5-year revision surgery rate after decompression alone for degenerative lumbar disease ranged broadly from 3.6% to 34.0% across 12 studies. A few studies conducted subanalysis on revision surgeries that were specifically conversions to fusion. Chan et al.^31^ reported that although 6.0% of patients in their study had revision surgery within 2 years after index decompression surgery, 2.4% (2 of 84 total patients) had a fusion procedure. Bydon et al.^32^ observed that the lifetime risk (average follow-up of 46.8 months) for requiring a fusion procedure after a lumbar laminectomy was 8.0%. Modhia et al.^33^ reported that the 2-year readmission rate for patients who underwent decompression alone for LSS was 13.2%, with 56% of the readmissions being for fusion procedures; thus, 7.4% of the decompression patients in their study experienced conversion to fusion. In the control group for our study, 3.8% of patients with LSS who underwent decompression alone as the index procedure required conversion to lumbar fusion after 5 years.

In the multivariate analysis, among a pooled group of the CES cohort and LSS control patients, CES was independently associated with increased 5-year risk of requiring a conversion to lumbar fusion. Although none of the other demographic and comorbidity risk factors were associated with increased risk of conversion to lumbar fusion, patients age 65 years and older and of male sex were observed to be independently associated with decreased risk of conversion to fusion. Similar to our findings, Keskimäki et al.^34^ reported that elderly patients underwent revision surgery at a decreased rate after lumber disk surgery. We hypothesize that this decreased rate of conversion to lumbar fusion in elderly patients is a reflection of risk stratification because a larger proportion of this patient cohort might be less suitable candidates for surgery. Our findings also align with a subanalysis of the Spine Patient Outcomes Research Trial, which found obesity, diabetes, and smoking status to have no notable association with revision surgery rate.^27^ However, neither of the aforementioned studies investigated conversion to fusion alone, instead considering risk factors associated with any revision surgery procedure. In addition, they did not include patients who underwent decompression due to CES, so our findings are difficult to compare. Ultimately, the most clinically notable finding from our multivariate analysis was that CES, independent of patient demographics and comorbidity risk factors, is associated with increased conversion to lumbar fusion with an OR of 2.13 (CI 1.56 to 2.97; *P* < 0.001).

While the subanalysis of how timing to decompression affected future conversion to lumbar fusion showed a higher trend in the patients treated within 48 hours compared with the patients treated between 48 hours and 10 days; ultimately, no statistical difference was observed between any of the timing groups. This result suggests that the emergent nature of decompression surgery for CES has minimal effect specifically on stability and risk of future conversion to fusion. Instead, we suspect that the increased rate of conversion to fusion in patients with CES compared with LSS control patients may be related to the technique of the decompression. Complete laminectomy and generous medial facetectomies may be necessary to achieve adequate decompression in some patients with CES. We speculate that this wide decompression, rather than emergent surgery, may be the driving factor for the results of our study; however, additional controlled investigation is needed to understand why this trend is observed.

There are several limitations to our study. First, with the use of a national insurance database, our study relies on the individual coding practices of physicians and hospitals. Because the nature of a national insurance database does not allow access to individual patient charts, this study depended on a coded diagnosis of CES, which may not have precisely correlated with the initial onset of symptoms if the patients were not properly diagnosed at the initial encounter; specifically, this may have added potential error to the subanalysis of time to decompression. In addition, the claims database does not capture the specific techniques used for decompression, such as full laminectomy versus partial hemilaminectomies, nor does it detail how much of the posterior elements were removed or preserved or how many levels were decompressed. Furthermore, although the fusion codes used in this study were specific for the lumbar region, we were not able to establish whether the conversion to fusion procedure occurred at the same level as the index decompression or how many levels were fused. However, we attempted to control for this through our matched analysis. Because both the CES cohort and LSS control group experienced this limitation, the absolute rates of conversion to fusion may have been affected, but the relative rates compared between the two groups still provide a clear demonstration of the increased risk of conversion to fusion among patients with CES. Finally, although this study examined patients who underwent decompression alone, some patients treated for CES undergo concomitant fusion in addition with decompression. The indications for fusion in CES are not clearly defined but may be associated with traumatic or iatrogenic instability.^20^ Although there is some limited evidence to suggest that fusion in addition to decompression may result in better outcomes,^35^ the increased cost and additional risk of instrumentation-related complications cloud this topic, and no trials to study this have been conducted. Our finding that patients with CES treated with decompression alone experience higher long-term rates of conversion to lumbar fusion warrants additional investigation into the indications for concomitant fusion and how the outcomes compare with decompression alone.

## Conclusion

Ultimately, our findings provide valuable information for counseling patients regarding risk for additional surgery. Compared with patients with LSS, patients who underwent decompression for CES were at increased risk of conversion to lumbar fusion. Because these surgeries come with increased morbidity and financial implications, setting realistic postoperative expectations after decompression surgery in CES is important for patient satisfaction and well-being.

## References

[1] Bagley CA Gokaslan ZL: Cauda equina syndrome caused by primary and metastatic neoplasms. Neurosurg Focus 2004;16:e3. 10.3171/foc.2004.16.6.3 15202873

[2] Kebaish KM Awad JN: Spinal epidural hematoma causing acute cauda equina syndrome. Neurosurg Focus 2004;16:e1. 15202871

[3] Harrop JS Hunt GE Vaccaro AR: Conus medullaris and cauda equina syndrome as a result of traumatic injuries: Management principles. Neurosurg Focus 2004;16:e4. 10.3171/foc.2004.16.6.4 15202874

[4] Jennett WB: A study of 25 cases of compression of the cauda equina by prolapsed intervertebral discs. J Neurol Neurosurg Psychiatry 1956;19:109-116. 13346384 10.1136/jnnp.19.2.109 PMC497193

[5] Kostuik JP: Medicolegal consequences of cauda equina syndrome: An overview. Neurosurg Focus 2004;16:e8. 10.3171/foc.2004.16.6.7 15202878

[6] Agarwal N Shah J Hansberry DR Mammis A Sharer LR Goldstein IM: Presentation of cauda equina syndrome due to an intradural extramedullary abscess: A case report. Spine J 2014;14:e1-e6. 10.1016/j.spinee.2013.09.029 24331844

[7] Lavy C James A Wilson-MacDonald J Fairbank J: Cauda equina syndrome. BMJ 2009;338:b936. 19336488 10.1136/bmj.b936

[8] Fraser S Roberts L Murphy E: Cauda equina syndrome: A literature review of its definition and clinical presentation. Arch Phys Med Rehabil 2009;90:1964-1968. 19887225 10.1016/j.apmr.2009.03.021

[9] Balasubramanian K Kalsi P Greenough CG Kuskoor Seetharam MP: Reliability of clinical assessment in diagnosing cauda equina syndrome. Br J Neurosurg 2010;24:383-386. 20726746 10.3109/02688697.2010.505987

[10] Gitelman A Hishmeh S Morelli BN : Cauda equina syndrome: A comprehensive review. Am J Orthop 2008;37:556-562. 19104682

[11] Ma B Wu H Jia LS Yuan W Shi GD Shi JG: Cauda equina syndrome: A review of clinical progress. Chin Med J (Engl) 2009;122:1214-1222. 19493474

[12] Spector LR Madigan L Rhyne A Darden B Kim D: Cauda equina syndrome. J Am Acad Orthop Surg 2008;16:471-479. 18664636 10.5435/00124635-200808000-00006

[13] Quaile A: Cauda equina syndrome-the questions. Int Orthop 2019;43:957-961. 30374638 10.1007/s00264-018-4208-0

[14] DeLong WB Polissar N Neradilek B: Timing of surgery in cauda equina syndrome with urinary retention: meta-analysis of observational studies. J Neurosurg Spine 2008;8:305-320. 18377315 10.3171/SPI/2008/8/4/305

[15] Ahn UM Ahn NU Buchowski JM Garrett ES Sieber AN Kostuik JP: Cauda equina syndrome secondary to lumbar disc herniation: A meta-analysis of surgical outcomes. Spine 2000;25:1515-1522. 10851100 10.1097/00007632-200006150-00010

[16] Shapiro S: Medical realities of cauda equina syndrome secondary to lumbar disc herniation. Spine 2000;25:348-351. 10703108 10.1097/00007632-200002010-00015

[17] Todd NV: Cauda equina syndrome: The timing of surgery probably does influence outcome. Br J Neurosurg 2005;19:301-306. 16455534 10.1080/02688690500305324

[18] Kohles SS Kohles DA Karp AP Erlich VM Polissar NL: Time-dependent surgical outcomes following cauda equina syndrome diagnosis: Comments on a meta-analysis. Spine 2004;29:1281-1287. 15167669 10.1097/00007632-200406010-00019

[19] Kennedy JG Soffe KE McGrAth A : Predictors of outcome in cauda equina syndrome. Eur Spine J 1999;8:317-322. 10483835 10.1007/s005860050180 PMC3611188

[20] Radcliff KE Kepler CK Delasotta LA : Current management review of thoracolumbar cord syndromes. Spine J 2011;11:884-892. 21889419 10.1016/j.spinee.2011.07.022

[21] Qureshi A Sell P: Cauda equina syndrome treated by surgical decompression: The influence of timing on surgical outcome. Eur Spine J 2007;16:2143-2151. 17828560 10.1007/s00586-007-0491-y PMC2140120

[22] Shapiro S: Cauda equina syndrome secondary to lumbar disc herniation. Neurosurgery 1993;32:743-746. 8492849 10.1227/00006123-199305000-00007

[23] McCarthy MJH Aylott CEW Grevitt MP Hegarty J: Cauda equina syndrome: Factors affecting long-term functional and sphincteric outcome. Spine 2007;32:207-216. 17224816 10.1097/01.brs.0000251750.20508.84

[24] Todd NV: Causes and outcomes of cauda equina syndrome in medico-legal practice: A single neurosurgical experience of 40 consecutive cases. Br J Neurosurg 2011;25:503-508. 21513452 10.3109/02688697.2010.550344

[25] Kara B Tulum Z Acar Ü: Functional results and the risk factors of reoperations after lumbar disc surgery. Eur Spine J 2005;14:43-48. 15490256 10.1007/s00586-004-0695-3 PMC3476671

[26] Sato S Yagi M Machida M : Reoperation rate and risk factors of elective spinal surgery for degenerative spondylolisthesis: Minimum 5-year follow-up. Spine J 2015;15:1536-1544. 25681581 10.1016/j.spinee.2015.02.009

[27] Gerling MC Leven D Passias PG : Risk factors for reoperation in patients treated surgically for lumbar stenosis: A subanalysis of the 8-year data from the SPORT trial. Spine 2016;41:901-909. 26656062 10.1097/BRS.0000000000001361 PMC5521164

[28] Thakur JD Storey C Kalakoti P : Early intervention in cauda equina syndrome associated with better outcomes: A myth or reality? Insights from the nationwide inpatient sample database (2005-2011). Spine J 2017;17:1435-1448. 28456676 10.1016/j.spinee.2017.04.023

[29] Lisai P Doria C Crissantu L : Cauda equina syndrome secondary to idiopathic spinal epidural lipomatosis. Spine 2001;26:307-309. 11224868 10.1097/00007632-200102010-00017

[30] Lang Z Li J-S Yang F : Reoperation of decompression alone or decompression plus fusion surgeries for degenerative lumbar diseases: A systematic review. Eur Spine J 2019;28:1371-1385. 29956000 10.1007/s00586-018-5681-2

[31] Chan AK Bisson EF Bydon M : Laminectomy alone versus fusion for grade 1 lumbar spondylolisthesis in 426 patients from the prospective Quality Outcomes Database. J Neurosurg Spine 2018;30:234-241. 30544348 10.3171/2018.8.SPINE17913

[32] Bydon M Macki M Abt NB : Clinical and surgical outcomes after lumbar laminectomy: An analysis of 500 patients. Surg Neurol Int 2015;6:S190-S193. 26005583 10.4103/2152-7806.156578 PMC4431053

[33] Modhia U Takemoto S Braid-Forbes MJ Weber M Berven SH: Readmission rates after decompression surgery in patients with lumbar spinal stenosis among Medicare beneficiaries. Spine 2013;38:591-596. 23324923 10.1097/BRS.0b013e31828628f5

[34] Keskimäki I Seitsalo S Osterman H Rissanen P: Reoperations after lumbar disc surgery: A population-based study of regional and interspecialty variations. Spine 2000;25:1500-1508. 10851098 10.1097/00007632-200006150-00008

[35] Dave BR Samal P Sangvi R Degulmadi D Patel D Krishnan A: Does the surgical timing and decompression alone or fusion surgery in lumbar stenosis influence outcome in cauda equina syndrome? Asian Spine J 2019;13:198-209. 30472822 10.31616/asj.2018.0168 PMC6454274

